# Identification of Constituents Affecting the Secretion of Pro-Inflammatory Cytokines in LPS-Induced U937 Cells by UHPLC-HRMS-Based Metabolic Profiling of the Traditional Chinese Medicine Formulation Huangqi Jianzhong Tang

**DOI:** 10.3390/molecules24173116

**Published:** 2019-08-27

**Authors:** Xuehong Nöst, Eva-Maria Pferschy-Wenzig, Stefanie Nikles, Xiaojuan He, Danping Fan, Aiping Lu, Jimmy Yuk, Kate Yu, Giorgis Isaac, Rudolf Bauer

**Affiliations:** 1 Department of Pharmacognosy, Institute of Pharmaceutical Sciences, University of Graz, Universitätsplatz 4/I, 8010 Graz, Austria; 2 School of Chinese Medicine, Hong Kong Baptist University, Kowloon Tong, Kowloon, Hong Kong, China; 3 Institute of Basic Research in Clinical Medicine, China Academy of Chinese Medical Sciences, Dongzhimennei Nanxiaojie 16, Beijing 100700, China; 4 Waters Corporation, 34 Maple Street, Milford, MA 01757, USA

**Keywords:** Huangqi Jianzhong Tang, *Astragalus mongholicus*, *Cinnamomum cassia*, *Glycyrrhiza uralensis*, *Paeonia lactiflora*, *Zingiber officinalis*, *Ziziphus jujube*, UPLC-QTOF-MS, metabolomics, TNF-α, IL-1β, IFN-γ

## Abstract

Within non-communicable diseases, chronic inflammatory conditions represent one of the biggest challenges for modern medicine. Traditional Chinese Medicine (TCM) has been practiced over centuries and has accumulated tremendous empirical knowledge on the treatment of such diseases. Huangqi Jianzhong Tang (HQJZT) is a famous TCM herbal formula composed of Radix Astragali, Ramulus Cinnamomi, Radix et Rhizoma Glycyrrhizae Praeparata cum Melle, Radix Paeoniae Alba, Rhizoma Zingiberis Recens, Fructus Jujubae and Saccharum Granorum (maltose), which has been used for the treatment of various chronic inflammatory gastrointestinal diseases. However, there is insufficient knowledge about its active constituents and the mechanisms responsible for its effects. The present study aimed at identifying constituents contributing to the bioactivity of HQJZT by combining in vitro cytokine production assays and LC-MS metabolomics techniques. From the HQJZT decoction as well as from its single herbal components, extracts of different polarities were prepared. Phytochemical composition of the extracts was analyzed by means of UPLC-QTOF-MS/MS. The inhibitory effects of the extracts on TNF-α, IL-1β and IFN-γ production were studied in U937 cells. Phytochemical and pharmacological bioactivity data were correlated by orthogonal projection to latent structures discriminant analysis (OPLS-DA) in order to identify those HQJZT constituents which may be relevant for the observed pharmacological activities. The investigations resulted in the identification of 16 HQJZT constituents, which are likely to contribute to the activities observed in U937 cells. Seven of them, namely calycosin, formononetin, astragaloside I, liquiritigenin, 18β-glycyrrhetinic acid, paeoniflorin and albiflorin were unambiguously identified. The predicted results were verified by testing these compounds in the same pharmacological assays as for the extracts. In conclusion, the anti-inflammatory activity of HQJZT could be substantiated by in vitro pharmacological screening, and the predicted activities of the OPLS-DA hits could be partially verified. Moreover, the benefits and limitations of MVDA for prediction pharmacologically active compounds contributing to the activity of a TCM mixture could be detected.

## 1. Introduction

During the last decades, the number of people suffering from non-communicable chronic diseases (NCDs) has increased dramatically all over the world. This phenomenon is related to enhanced lifespan on the one hand, but also to modern lifestyle and environmental factors, including unhealthy diet, lack of exercise, stress, smoking, air pollution etc., on the other hand. Within NCDs, chronic inflammatory conditions represent one of the biggest challenges for modern medicine, and new therapeutic approaches are in great demand to sustain or improve the quality of life of the patients [1,2]. Traditional Chinese Medicine (TCM) has been practiced over centuries in Asia and relies on tremendous empirical knowledge. In contrast to Western medicine, TCM describes the body and its malfunctions as a holistic system. Therefore, the therapy does not only focus on the symptoms but is directed towards the causes and the patient´s whole body. Decoctions are one of the most common therapeutic application forms in TCM. By combination of different herbs, minerals, and animal products, synergistic effects may be achieved and side effects reduced [3].

Huangqi Jianzhong Tang (HQJZT) is a famous TCM formula composed of seven ingredients: Radix Astragali (RA, Huangqi), Ramulus Cinnamomi (RC, Guizhi), Radix et Rhizoma Glycyrrhizae Praeparata cum Melle (RRGP, Zhigancao), Radix Paeoniae Alba (RP, Baishao), Rhizoma Zingiberis Recens (RZ, Shengjiang), Fructus Jujubae (FJ, Dazao), and Saccharum granorum (Yi Tang). It derives from the TCM classic “Jin Gui Yao Lue” (*Essential Prescriptions from the Golden Cabinet*) and is traditionally used for the treatment of chronic gastrointestinal diseases such as chronic gastritis, peptic ulcers, inflammatory bowel disease, autonomic dystonia, chronic hepatitis, and chronic nephritis [4,5]. Apart from this, in a clinical study, the formulation showed effects in reducing fatigue in athletes by increasing the oxygen uptake and the systemic oxygen supply [6]. There is some evidence from animal experiments and human clinical studies substantiating the use of HQJZT for the treatment of chronic gastritis and irritable bowel syndrome (IBS). These studies indicate that the effects of the formula might be based on immunomodulatory, anti-inflammatory and anti-oxidative effects, and on epithelial and mucosal protection, suggesting the formula as a multi-target-directed agent [7,8,9]. However, there is insufficient knowledge about the active constituents responsible for the beneficial effects of HQJZT.

The anti-inflammatory activity of HQJZT was studied by screening for inhibitory effects on the production of the pro-inflammatory cytokines TNF-α, IL-1β and IFN-γ in U937 human leukemic cells. Cytokines are small proteins secreted by cells which play a fundamental role in cell signaling and intercellular communication. They are key mediators in a wide range of biological processes, including cell growth and migration, innate and acquired immunity, inflammation etc. [10,11]. Nearly all cell types can release cytokines, although the two principal producers are helper T cells (Th cells) and macrophages. The family of cytokines comprises a broad range of molecules that possess different structures and exhibit diverse functions (e.g., interferons (IFNs), interleukins (ILs), chemokines, tumor necrosis factors (TNFs) and colony stimulating factors) [12]. The cytokines of interest chosen for this study (TNF-α, IL-1β and IFN-γ) are pro-inflammatory cytokines. They are predominantly produced in activated macrophages and involved in the up-regulation of inflammatory reactions [12].

Metabolomics is a relatively new “omics” technique allowing high throughput qualitative and quantitative analysis of all small molecules present in a biological sample [13]. The outcome of these analyses are usually complex multi-dimensional datasets demanding for multivariate data analysis. Both unsupervised methods (e.g. principal component analysis (PCA)) and supervised methods (e. g. orthogonal projection to latent structures (OPLS) and their formulation for discriminant analysis (DA)) can be applied for multivariate data analysis [14]. Due to the complexity of herbal extracts like they are used in TCM, the currently most widespread methods for their analysis are nuclear magnetic resonance (NMR) spectroscopy or mass spectrometry (MS), the latter one usually preceded by chromatographic separation steps such as gas (GC) or liquid chromatography (LC) and in particular ultra-high performance liquid chromatography (UHPLC) [15]. Hyphenated with high resolution MS, UHPLC allows fast and direct separation of crude plant extracts with high sensitivity and reproducibility and provides detailed ion maps of all detectable features in the sample [16]. UHPLC-HRMS untargeted metabolomics can be used for a broad range of purposes in NPs research, such as the general investigation of the chemical composition of herbal extracts or also to discriminate metabolite variations in plants grown under different conditions [17]. By applying metabolomics, novel substances have been discovered and structure-bioactivity relationships have been detected, leading to the identification of active leads without prior isolation. The technique is also able to assess bioavailability, metabolism, safety and toxicity of herbal medicines in human body [13]. The combination of NMR- or MS- based metabolomics with supervised multivariate data analysis methods has been successfully applied in NPs research: For example, OPLS-DA has been used to identify discriminant markers in the ^1^H-NMR and UHPLC-MS metabolic profiles of the closely related species *Pelargonium sidoides* and *P. reniforme* [18]; partial least squares discriminant analysis (PLS-DA) of UHPLC-TOF-MS data has been applied for the identification of chemical quality markers for different *Ficus deltoidea* varieties [19]. Correlating bioactivity data with metabolomic data has been successfully used to predict bioactive plant constituents which contribute to the activity of herbal extracts: For example, PLS-DA was used to predict the bioactive principles from ^1^H-NMR metabolomic data of *Galphimia glauca* accessions with distinct in vivo sedative and anxiolytic activities [20]; recently, compounds with anti-biofilm activity were identified by correlating the LC-MS profiles of six marine *Streptomyces* strains with bioactivity data by means of PLS-DA [21].

The aim of the present study was to correlate UHPLC-HRMS metabolic profiles of different sub-extracts of the complex TCM formula HQJZT with screening data from cellular in vitro assays on cytokine production in order to identify constituents, which may contribute to the pharmacological effects of HQJZT.

## 2. Results

### 2.1. Fractions of HQZT Decoction and Its Single Herbs Exert Distinct Effects on Pro-Inflammatory Cytokines

Decoctions of the whole HQJZT formula and each contained single herbal component were prepared and fractionated by liquid-liquid extraction (LLE) using solvents of different polarities. From these sub-extracts, the dichloromethane (DCM), ethyl acetate (EtOAc) and *n*-butanol (*n*-BuOH) sub-extracts were included in the pharmacological screening. Testing of *n*-hexane extracts was not possible due to their extremely low extract yields (see Appendix A, Table A1). The residual aqueous phases also were not considered for pharmacological testing, since according to the results of preliminary LC-MS experiments, they mainly contained very hydrophilic compounds such as sugars which were supposed to be irrelevant for pharmacological effects since they are usually degraded in vivo in the gastrointestinal tract (see Appendix A, Figure A2).

**Figure A1:**
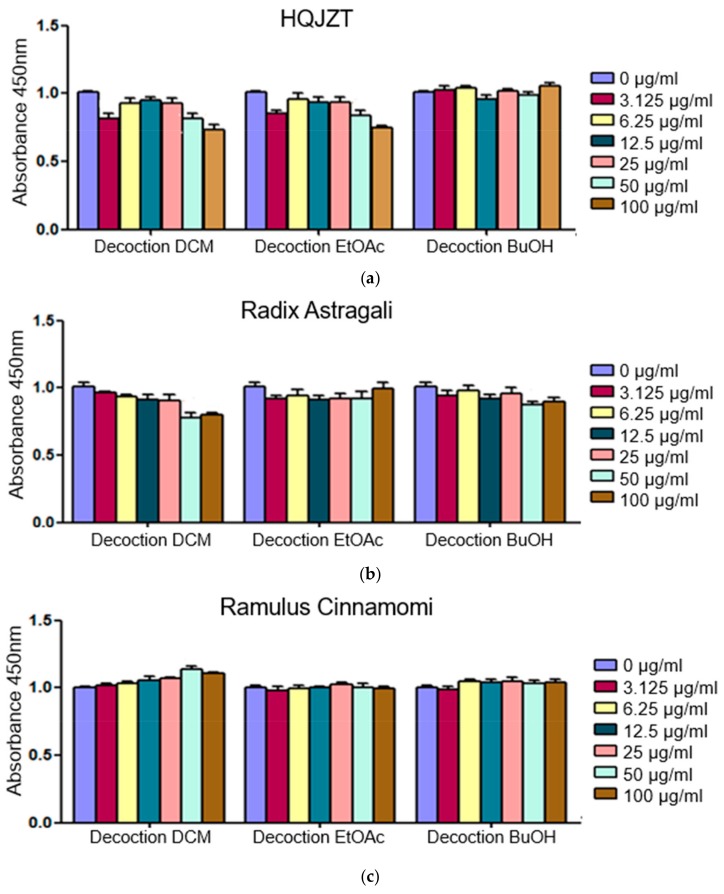

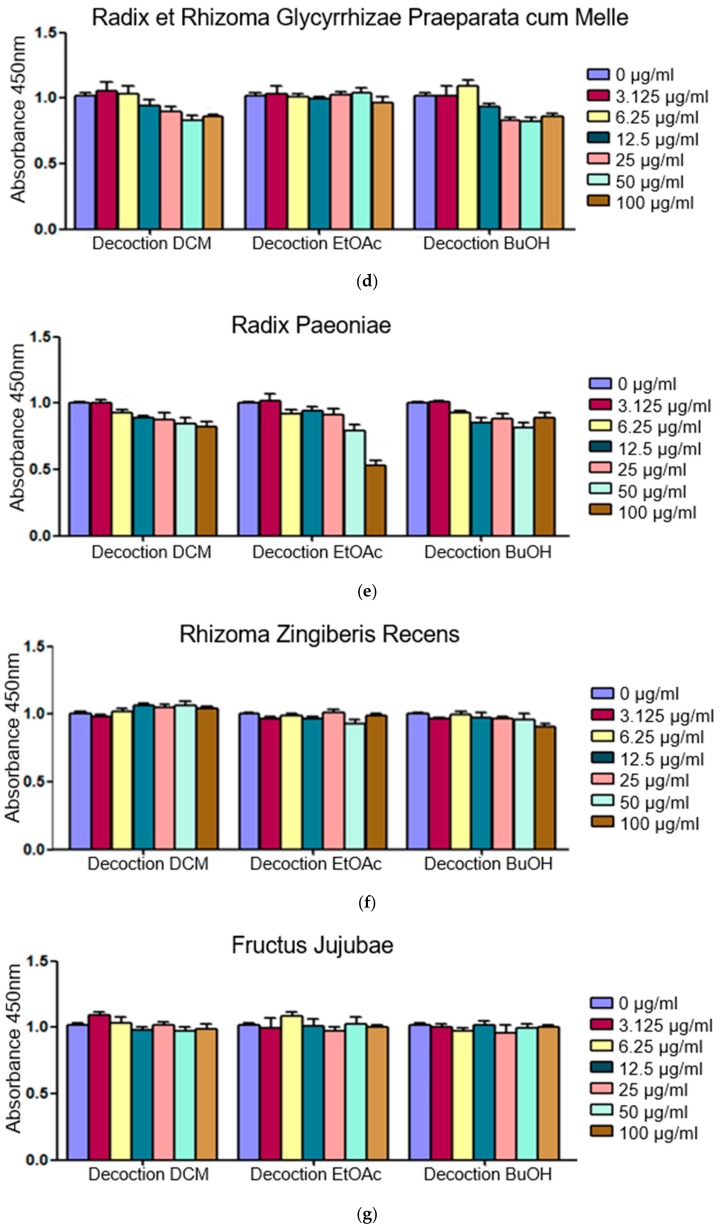
Effects of the extracts from the decoctions on cell viability; (**a**) HQJZT; (**b**) RA; (**c**) RC; (**d**) RRGP; (**e**) RP; (**f**) RZ; (**g**) FJ; n = 3; mean (SD).

**Figure A2:**
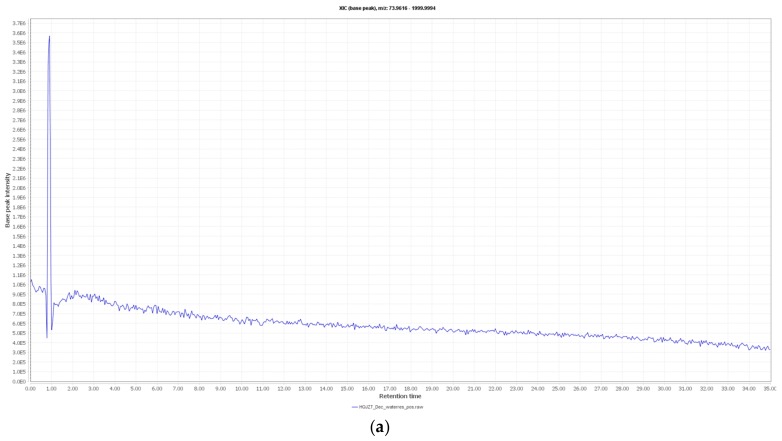

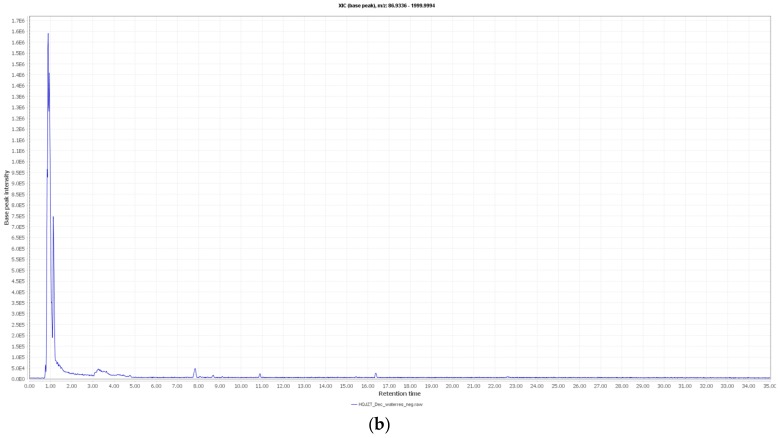
UHPLC-HRMS chromatograms of HQJZT water residue sample in ESI (+) mode (**a**) and ESI (−) mode (**b**); The main peak at 0.86 min was identified as sucrose. Analysis was performed on an Ultimate 3000 UHPLC system (LC parameters as described in the Materials and methods section) hyphenated with an LTQ-XL linear ion trap mass spectrometer (Thermo Scientific, Waltham, MA, USA).

In order to determine the non-toxic concentration of the extracts to be used in the subsequent tests on inhibitory activity on production of cellular TNF-α, IL-1β and IFN-γ, the CKK-8 cytotoxicity assay was performed as a first step. The results are shown in Figure A1. At a concentration of 25 μg/mL, none of the test samples showed any cellular toxicity after 24 hours, therefore, this concentration was chosen as test concentration for the cytokine production assays.

**Figure 1:**
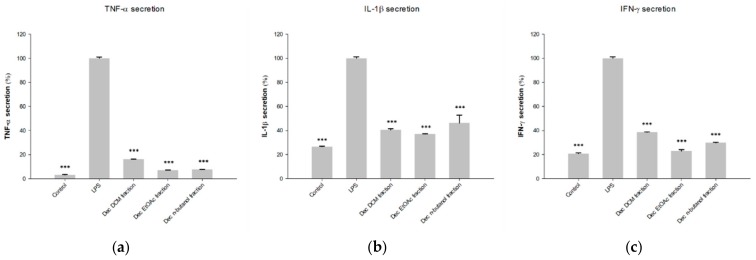
Inhibitory effects of the LLE fractions of the decoction of HQJZT on production of (**a**) TNF-α; (**b**) IL-1β and (**c**) IFN-γ; concentration of the extracts: 25 µg/mL; n = 3; mean (SD); Control: unstimulated cells; LPS: LPS-stimulated cells (1 µg/mL) without treatment; * (*p* < 0.05); ** (*p* < 0.01); *** (*p* < 0.005); significant differences obtained by ANOVA with Dunnett-T post-hoc.

**Figure 2:**
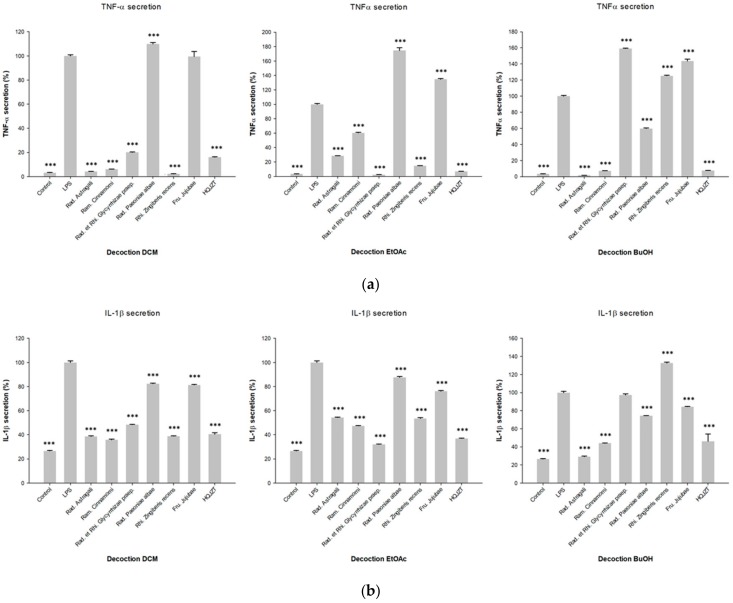

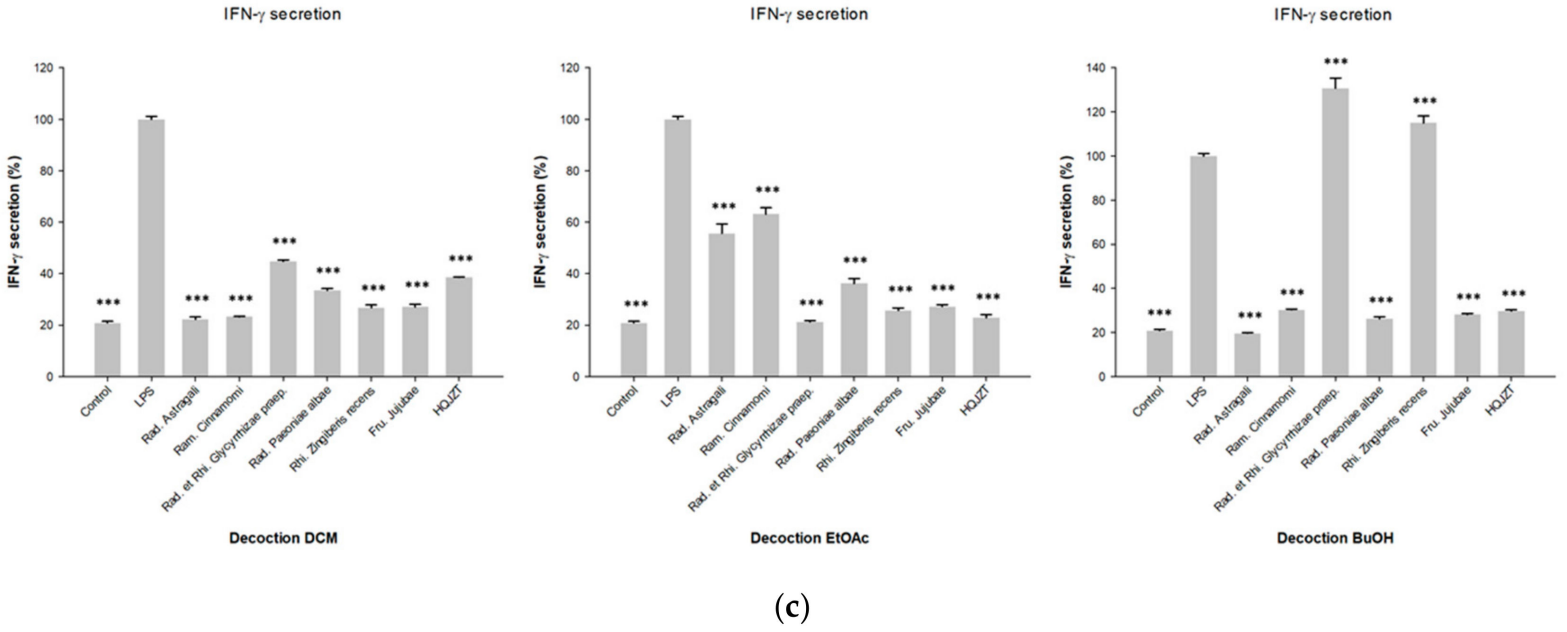
Inhibitory effects of the fractions of the decoctions of HOJZT and its herbal components on production of (**a**) TNF-α; (**b**) IL-1β and (**c**) IFN-γ; concentration of the extracts: 25 µg/mL; n = 3; mean (SD); Control: unstimulated cells; LPS: LPS-stimulated cells (1 µg/mL) without treatment; * (*p* < 0.05); ** (*p* < 0.01); *** (*p* < 0.005); significant differences obtained by ANOVA with Dunnett-T post-hoc.

The inhibitory activities of the fractions produced by LLE of HQJZT decoction against the production of pro-inflammatory cytokines TNF-α, IL-1β and IFN-γ are shown in Figure 1. All HQJZT fractions exhibited significant activities in all three assays, indicating that the formulation possesses in vitro anti-inflammatory activity. The fact that polar as well as non-polar extracts showed pronounced activities indicates that several constituents with different polarities are involved in the observed effects. The LLE fractions of the single HQJZT components showed more distinct effects towards the levels of the three tested cytokines, as shown in Figure 2.

In the assay on TNF-α production, most pronounced activities were observed for all three fractions from Radix Astragali, as well as for the DCM and EtOAc fractions of Radix Glycyrrhizae Praeparata and Rhizoma Zingiberis recens, and for the DCM and *n*-BuOH fractions of Radix Paeoniae Alba. The other fractions showed weaker or no activity, or even led to enhanced TNF-α levels (Figure 2a).

Reduction of IL-1β levels was most pronounced for the DCM and n-BuOH fractions of Radix Astragali and Ramulus Cinnamomi, the EtOAc fraction of Radix Glycyrrhizae Praeparata and for the DCM fraction of Radix Zingiberis recens (Figure 2b).

Regarding the inhibition of LPS-induced IFN-γ secretion, all fractions from Radix Paeoniae Alba and Fructus Jujubae showed pronounced activity. The DCM and EtOAc fractions from Radix Glycyrrhizae Praeparata and from Rhizoma Zingiberis, as well as the DCM and n-BuOH fractions of Radix Astragali and Ramulus Cinnamomi also showed pronounced inhibitory activity in this assay (Figure 2c).

### 2.2. Fractions of HQZT Decoction and Its Single Herbs Possess Distinct UPLC-HRMS Profiles

**Figure 3:**
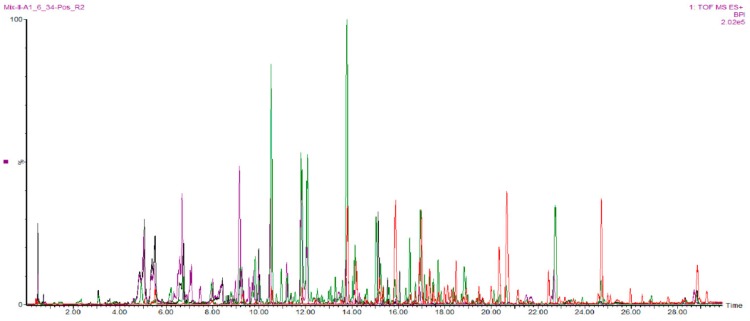
Base peak UPLC-QTOF-MS chromatograms of HQJZT fractions in ESI positive ion mode; red: *n*-hexane fraction, green: DCM fraction, violet: EtOAc fraction, black: *n*-BuOH fraction.

In our study, UPLC was used in order to obtain a good separation of the complex mixture of constituents present in the HQJZT fractions. The QTOF-MS instrument was run in the ESI positive mode which allowed to detect a wide range of constituents of HQJZT (Figure 3).

**Figure 4:**
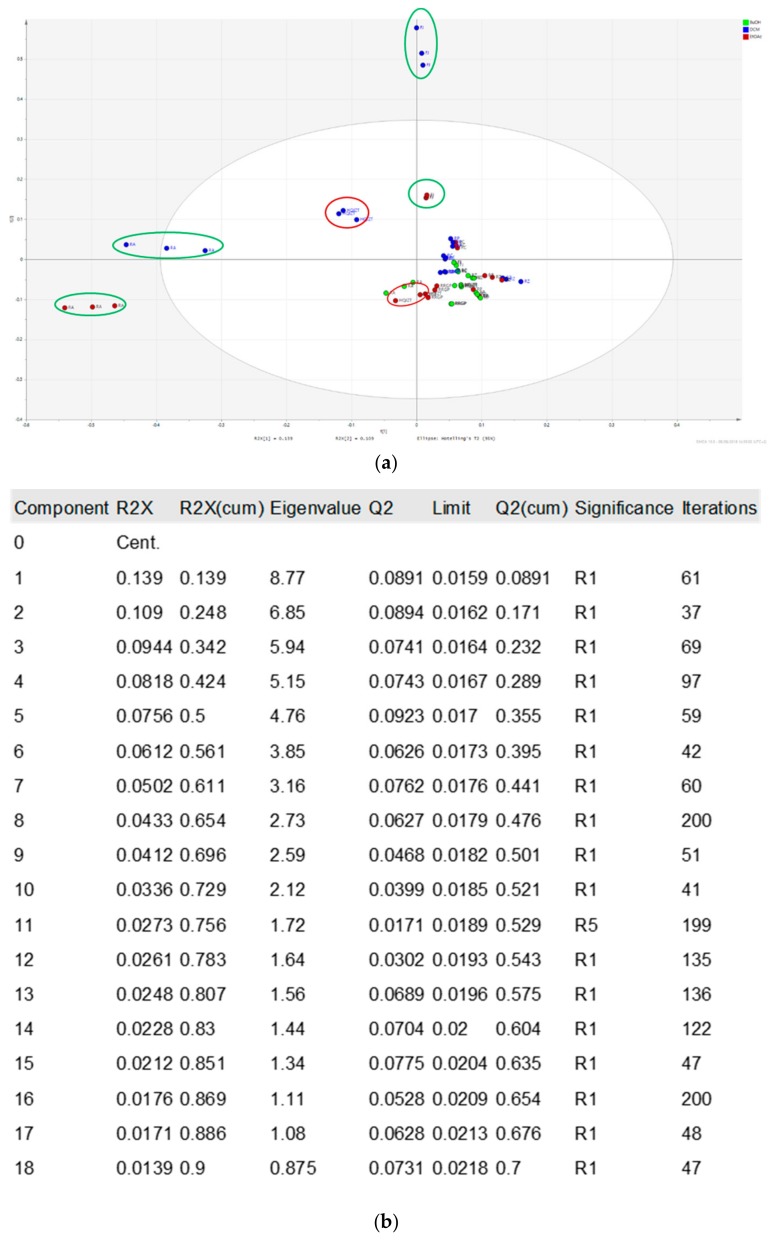
PCA t[1]/t[2] Score scatter plot of the UPLC-QTOF-MS data of all pharmacologically tested samples from Huangqi Jianzhong Tang (green: n-BuOH fractions; blue: DCM fractions; red: EtOAC fractions) (**a**) PCA plot; (**b**) model window.

The PCA score scatter plot (Figure 4) of all tested fractions obtained from HQJZT and its single herbal components shows that the non-polar fractions (DCM and EtOAc) from Fructus Jujubae and Radix Astragali (marked in green circles) display the biggest differences compared to the DCM and EtOAc extracts of HQJZT decoction (marked in red circles). For the other herbal components, there were no significant differences between fractions produced with different solvents.

**Figure 5:**
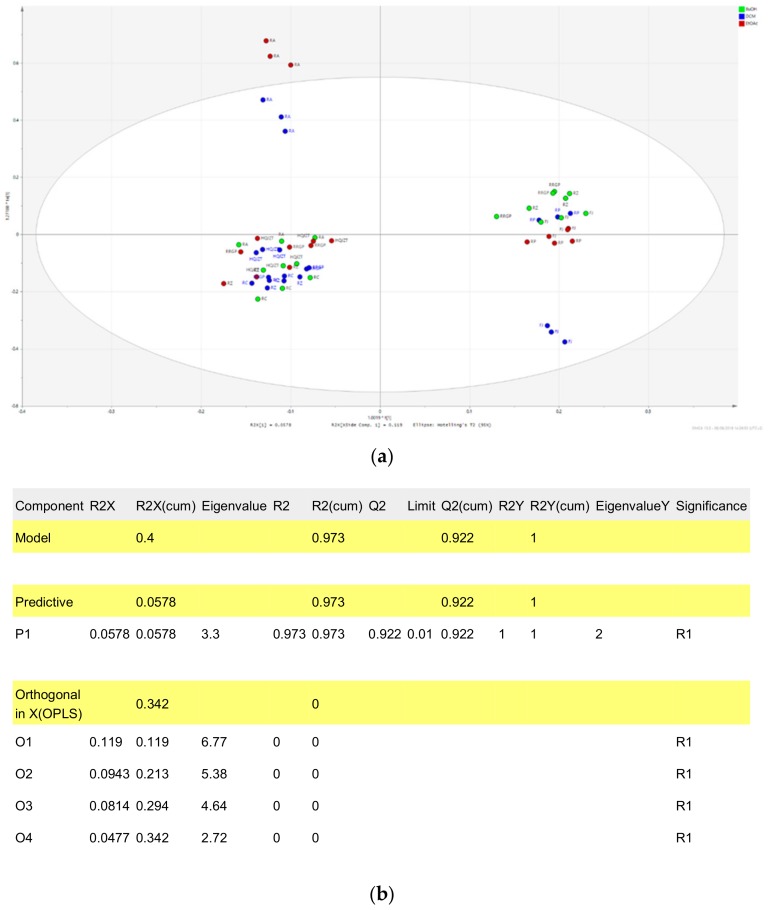

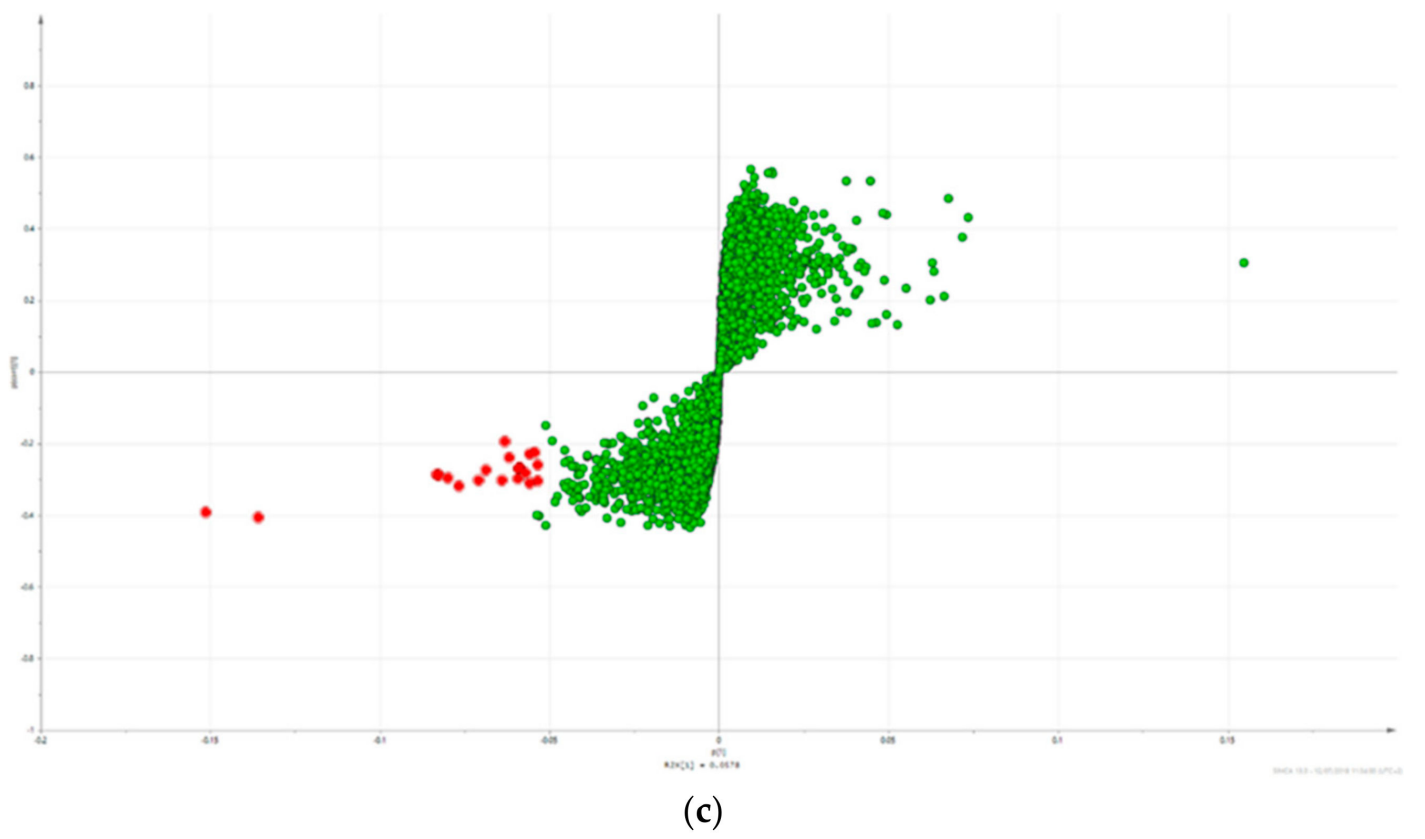
OPLS-DA model using the data from the UPLC-QTOF-MS and pharmacological analysis on inhibition of TNF-α production (active vs. inactive extracts). (**a**) t[1]/t0[1] Score scatter plot; (**b**) model window; (**c**) S-plot; Hit compounds are marked in red.

**Figure A3:**
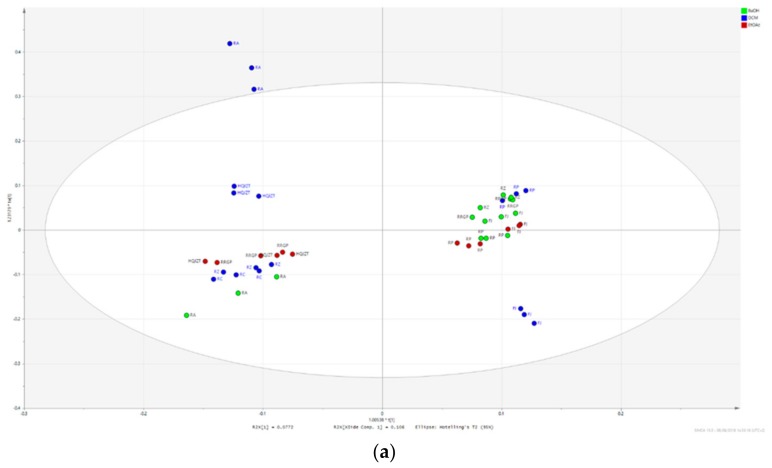

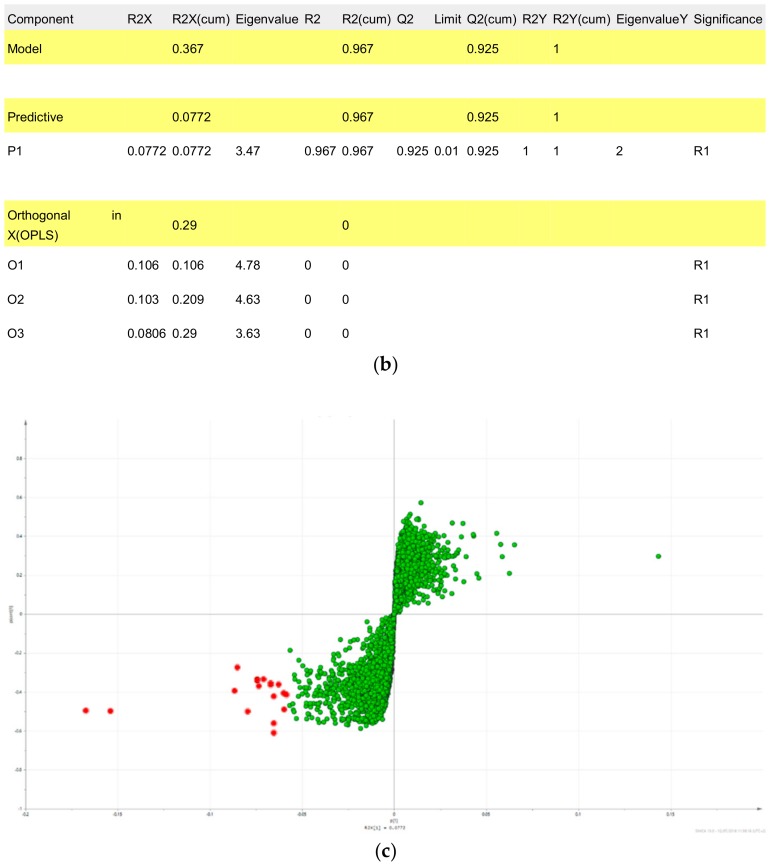
OPLS-DA model using the data from the UPLC-QTOF-MS and pharmacological analysis on inhibition of IL-1β production (active vs. inactive extracts). (**a**) t[1]/t0[1] Score scatter plot; (**b**) model window; (**c**) S-plot; Hit compounds are marked in red.

**Figure A4:**
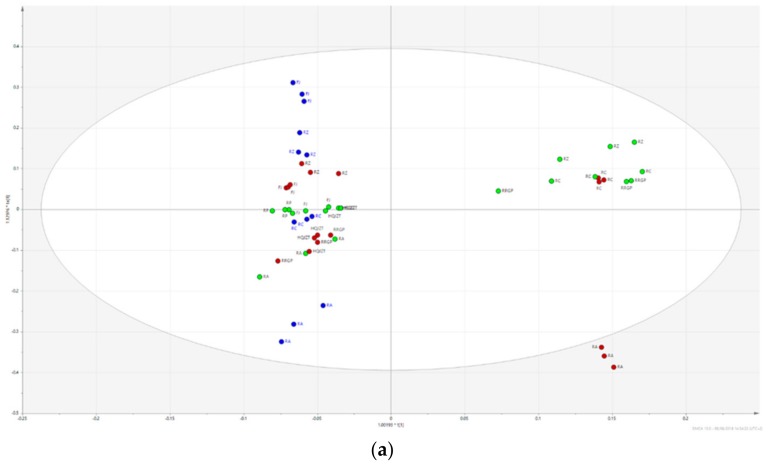

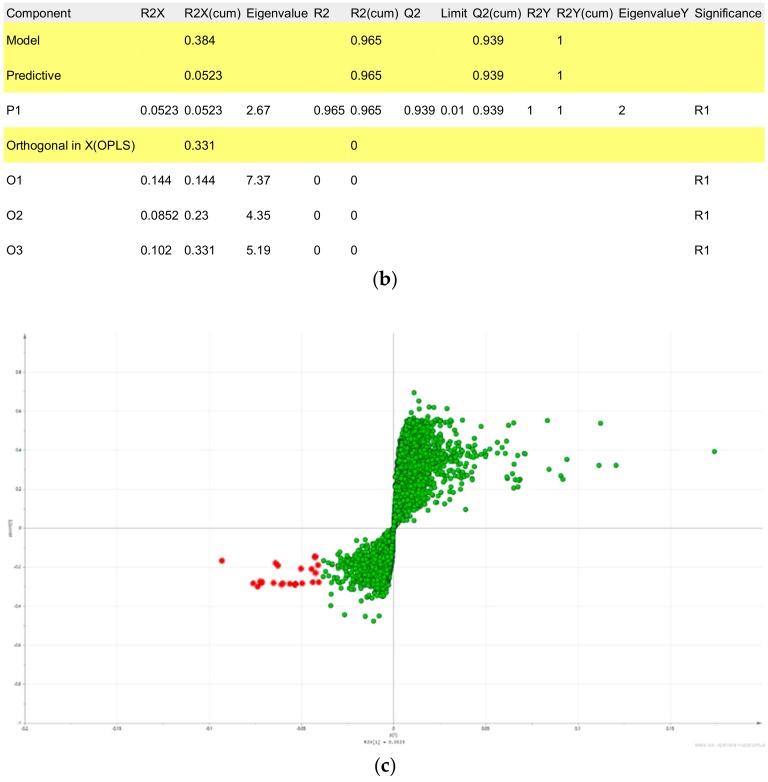
OPLS-DA model using the data from the UPLC-QTOF-MS and pharmacological analysis on inhibition of IFN-γ production (active vs. inactive extracts). (**a**) t[1]/t0[1] Score scatter plot; (**b**) model window; (**c**) S-plot; Hit compounds are marked in red.

In order to be able to predict constituents responsible for the anti-inflammatory activities of HQJZT, UPLC-HRMS data were correlated to bioactivity screening data by means of OPLS-DA, a supervised multivariate data analysis method [13]. For generating the OPLS-DA models, results from each bioassay were separated into three classes, i.e. “active”, “moderately active”, and “inactive” (Table 1), and the samples from the two classes “active” and “inactive” were included in the respective OPLS-DA models. Serious outliers were identified on the basis of Hotelling’s T2 range line plot and excluded from the model. t[1]/t0[1] Score scatter plots of the three models (see Figure 5 and Figure A3 and Figure A4a ) indicate a good separation between active and inactive samples. The quality of the models is displayed in the respective model window (see Figure 5 and Figure A3 and Figure A4b ).

The compounds most likely contributing to the observed pharmacological effects (hits) were identified on the basis of the OPLS-DA S-plot as shown in Figure 5 and Figure A3 and Figure A4c (18–20 hits for each tested activity, see Table A2, Table A3 and Table A4).

### 2.3. Triterpene Glycosides, Flavonoids and Monoterpene Glycosides are Most Strongly Correlated with Inhibitory Activity on Production of Pro-Inflammatory Cytokines

Targeted library search in the TCM library integrated in UNIFI as well as manual comparison of molecular formulas and fragmentation patterns with data from literature and public databases (Metlin, HMDB) allowed tentative identification of 16 of the hit compounds suggested by OPLS-DA (Table 2). The identity of seven of these compounds was subsequently unambiguously determined by UHPLC-MS comparison with authentic reference compounds.

### 2.4. Predicted Pharmacological Activities Can Be Partially Verified for the Identified OPLS-DA Hit Compounds

In order to validate the predictions achieved from supervised multivariate data analysis, the unambiguously identified hit compounds, namely astragaloside I astragaloside II, calycosin, formononetin, liquiritigenin, paeoniflorin, and albiflorin, were tested again as pure compounds in in vitro cellular assays on inhibition of pro-inflammatory cytokines, using the same experimental protocol as for the fractions. In addition, isoliquiritigenin was also included in the pharmacological testing. Isoliquiritigenin was not listed as one of the 18-20 OPLS-DA hits, however, it is a structural isomer of the OPLS-DA hit liquiritigenin and was therefore considered as an interesting test compound for verification of OPLS-DA prediction.

Prior to testing them in the cytokine production assays, the pure hit compounds were also subjected to cytotoxicity screening in the CCK8-assay in order to determine a non-toxic test concentration. According to the results of the CCK-8 assay (Figure A6), a test concentration of 6.25 µg/mL was chosen for the assays on cellular TNF-α, IL-1β and IFN-γ production.

**Figure 6:**
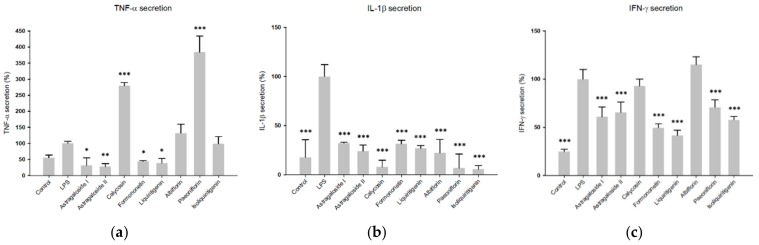
Inhibitory effects of the identified compounds in HQJZT on production of (**a**) TNF-α, (**b**) IL-1β and (**c**) IFN-γ. Concentration of the compounds: 6.25 µg/mL; n = 3; mean (SD); Control: unstimulated cells; LPS: LPS-stimulated cells (1 µg/mL) without treatment; * (*p* < 0.05); ** (*p* < 0.01); *** (*p* < 0.005); significant differences obtained by ANOVA with Dunnett-T post-hoc.

Regarding the inhibition of TNF-α production in LPS-induced U937 cells, astragaloside I and II, formononetin and liquiritigenin showed remarkable effects. At the test concentration of 6.25 µg/mL, they reduced TNF-α production by 68.6%, 72.1%, 55.7% and 60.9%, respectively. These results are in accordance with predictions derived from the OPLS-DA model. However, although predicted by the OPLS-DA model, calycosin did not show any inhibitory activity against TNF-α production at the screening concentration (see Figure 6a). The triterpene glycosides astragaloside I and II, as well as the isoflavone formomonetin, are constituents of Radix Astragali, while calycosin is an isoflavone with a very similar structure to formomonetin that is contained in Radix et Rhizoma Glycyrrhizae Praeparata. Albiflorin and paeoniflorin were also tested in this assay, although they were predicted to be only active against IFN-γ production, and in accordance with prediction, no effects of these compounds were observed in the TNF-α production assay at a concentration of 6.25 µg/mL.

**Figure A5:**
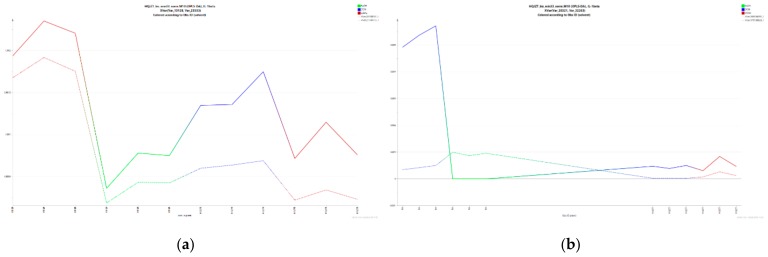
XVar Plots illustrating the levels of the compounds (**a**) liquiritigenin (257.08161_9.822) and isoliquiritigenin (257.08155_13.588) in Radix et rhizoma glycyrrhizae preparata (RRGP) extracts and in HJQZT extract, and (**b**) astragaloside I (1737.96629_18.398) and astragaloside II (1653.94391_15.879) in Radix Astragali (RA) and in HQJZT extracts.

**Figure A6:**
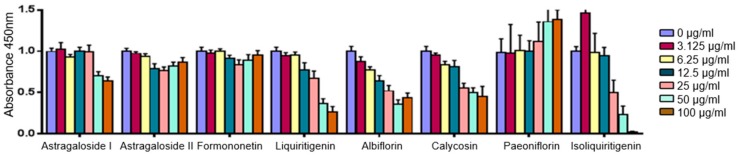
Effects of the pure compounds on cell viability; n = 3; mean (SD).

All tested compounds significantly reduced the secretion of IL-1β, with calycosin showing the most pronounced effect (reducing IL-1β secretion by 92.7% at the test concentration of 6.25 µg/mL), which was predicted by MVDA. The other predicted active compounds liquiritigenin, astragaloside I and formononetin showed inhibition rates of 73.1%, 67.9% and 68.5% at this concentration, respectively. Astragaloside II and isoliquiritigenin also reduced IL-1β secretion, although they were not predicted to be active by the OPLS-DA model. However, compared to the structurally related compounds Astragaloside I and liquiritigenin, their levels detected in their respective single extracts and in HQJZT are much lower, possibly leading to false negative prediction in the OPLS-DA model (Figure A5). Interestingly, paeoniflorin and albiflorin strongly reduced IL-1β production, although the extracts obtained from Radix Paeoniae Alba did not show strong activity in this assay and although paeoniflorin and albiflorin were not predicted as bioactives (see Figure 6b). This may possibly be caused by a discrepancy between test concentrations used for the pure compounds and compound levels present in the tested extracts.

Concerning the OPLS-DA model for IFN-γ inhibition, we were able to identify only two components potentially responsible for activity, since in the OPLS-DA hitlist, many of the hit features turned out to be various adducts or dimers of these two compounds. Indeed, a reduction of IFN-γ production was observed for pure paeoniflorin (−29.2%), however, in contrast to OPLS-DA prediction, its structural isomer albiflorin did not reduce, but even enhance IFN-γ-production.

Surprisingly, liquiritigenin (−58.4%), formononetin (−50.4%), isoliquiritigenin (−42.1%), astragaloside I (−39.1%) and astragaloside II (−34.4%) exerted inhibition against IFN-γ production at the screening concentration, although they had not been predicted as bioactive by the OPLS-DA model (see Figure 6c). A summary of all tested pure compounds, their predicted bioactivities and effectively verified activities is shown in Table A5.

## 3. Discussion

Since HQJZT is a very complex mixture containing a broad range of phytochemicals from diverse classes of compounds, liquid-liquid extraction of the decoctions of HQJZT and its individual herbs with solvents of increasing polarity was performed in order to allow selective enrichment of constituents, which may contribute to the activity, according to polarity. Highest yields were obtained for all samples after liquid-liquid extraction with *n*-butanol, while the amounts were very low for *n*-hexane sub-extracts. These results were in accordance with our expectations, since the initial extracts before liquid-liquid fractionation were decoctions which are prepared by boiling with water. With this extraction protocol, the polar components of the herbs were primarily extracted while lipophilic compounds were only extracted to a very low degree.

Preliminary LC-MS analyses suggested that in the ESI positive mode, more HQJZT constituents could be detected than in the negative mode, which showed no major additional peaks (data not shown). Therefore, positive mode was chosen for analysis of all samples.

The results from in vitro pharmacological screening indicate that the formula HQJZT has in vitro anti-inflammatory properties by inhibiting the release of the cytokines TNF-α, IL-1β and IFN-γ from macrophages, and that the different herbal components of the mixture contribute to the observed activities to a different extent.

TNF-α is a multifunctional cytokine primarily produced by immune cells such as monocytes and macrophages and plays a key role in apoptosis, cell survival, immunity, and inflammation [27,28]. It is generated as a precursor form—transmembrane TNF-α, which transforms to the soluble form by processing by the TNF-α-converting enzyme (TACE) and mediates its biological activities via two receptors, TNFR-1 and TNFR-2 [29]. There is considerable evidence suggesting a relationship between overproduction of TNF-α and chronic inflammatory diseases such as rheumatoid arthritis, psoriatic arthritis, psoriasis, or inflammatory bowel disease. Therefore, various strategies have been developed to neutralize TNF-α, including antibodies, soluble receptors, recombinant TNF binding proteins, etc. [27,30].

IL-1β belongs, together with ten other members, to the IL-1 family. It can be produced and secreted by a variety of different cell types, like monocytes and macrophages, but also by nonimmune cells, such as fibroblasts, neutrophils and endothelial cells, in response to molecular motifs carried by pathogens called “pathogen associated molecular patterns” (PAMPs) [12,31]. These stimuli further lead to the release of the inactive precursor form called pro-IL-1β, which has no biological effect until it is cleaved by caspase-1. IL-1β exerts its various potentiating effects on cell proliferation, differentiation, and function of many innate and specific immune cells by binding to the IL-1 type receptors and is correlated to a wide range of autoimmune and inflammatory diseases, such as rheumatoid arthritis, irritable bowel syndrome, and psoriasis [32].

IFN-γ is selectively produced by immune cells (natural killer cells, T lymphocytes, and NKT cells) while its receptor (IFNGR) can be found in nearly all cell types [33,34]. Its secretion is controlled by cytokines secreted by antigen presenting cells (APCs) in response to infections [35]. Once bound to its receptor, IFN-γ can induce anti-viral, anti-proliferative and immunomodulatory effects in both, pro- and anti-inflammatory directions. This regulatory ability is the key to the balance of our immune system as IFN-γ is able to synergize and to antagonize the effects of cytokines, growth factors, and PAMP signaling pathways [35,36]. There is some controversial evidence regarding the beneficial and pathogenic effects of IFN-γ in development and therapy of autoimmune and inflammatory diseases, such as systemic Lupus erythematodes, multiple sclerosis, rheumatoid arthritis, and arteriosclerosis [34,37,38].

Two HJQZT constituents that seem most likely involved in the inhibition of both TNF-α and IL-1β production according to the respective OPLS-DA models were unambiguously identified as calycosin and formononetin. Both compounds are isoflavonoids contained in Radix Astragali. Total flavonoids of *Astragalus mongholicus* have already been reported to possess immunomodulatory activities through bidirectional modulation of cytokine release in RAW 264.7 macrophages next to various other anti-inflammatory effects in vivo [39]. Calycosin has been shown to suppress the mRNA expression of various pro-inflammatory cytokines via the activation of p62/Nrf2-linked heme oxygenase 1 in vitro [40]. Li et al. isolated twelve *Astragalus mongholicus* flavonoids and demonstrated their inhibitory effect on LPS-induced overproduction of pro-inflammatory cytokines IL-6 and IL-12 p40 in bone marrow derived dendritic cells [41].

According to the OPLS-DA model, the triterpene saponins astragalosides I and II should also exert immunomodulatory effects. Astragaloside I was among the hits for both TNF-α and IL-1β inhibition, astragaloside II is supposed to inhibit TNF-α production specifically. While there are some works focusing on the immunomodulatory activity of astragaloside IV, only one study including astragaloside I and II could be found [42].

Liquiritigenin, a flavonoid from Radix et Rhizoma Glycyrrhizae Praeparata, was predicted to inhibit IL-1β production, and to a smaller extent, also TNF-α secretion. The anti-inflammatory activity of licorice and its constituents has already been described by many authors. Yu et al. demonstrated the anti-inflammatory activities of licorice extract and its active compounds, glycyrrhizic acid, liquiritin, and liquiritigenin in BV2 cells and mice liver [43]. Kim et al. showed that the anti-inflammatory effects of liquiritigenin are due to the inhibition of NF-κB-dependent iNOS and pro-inflammatory cytokine production [44].

Polar constituents from Radix Paeoniae Alba seem to be primarily responsible for the inhibitory activity of HQJZT on IFN-γ release. Two of them were identified as paeoniflorin and albiflorin by comparing retention time and fragmentation pattern with reference substances. Wang and co-workers proved that total glucosides of peony (TGP) can decrease serum levels of IFN-γ and other pro-inflammatory cytokines in patients with psoriatic arthritis in a clinical trial [45]. TGP were also shown to attenuate TNBS-induced colitis in rats by decreasing up-regulated pro-inflammatory cytokines TNF-α and IL-1β, and increasing down-regulated anti-inflammatory cytokine IL-10 [46]. Other studies demonstrated the beneficial effect of Radix Paeoniae on arthritis by inhibiting the proliferation of synoviocytes, leading to decreased production of pro-inflammatory cytokines [47,48]. The anti-inflammatory activity and structure-activity relationship of nine monoterpene derivatives from Radix Paeoniae in LPS-stimulated RAW 264.7 cells were described by Bi et al. [49]. Unfortunately, most of the mentioned work did not specifically focus on IFN-γ production. So, obviously more investigations in this field are required.

On the basis of these findings, seven of the identified compounds, together with isoliquiritigenin, were tested again in the same in vitro cell assay in order to verify the predicted results. The results with the pure compounds revealed that at the screening concentration of 6.25 µg/mL, formononetin reduced both, TNF-α and IL-1β secretion, by more than 50%, which confirmed the prediction of the statistical model. Calycosin specifically reduced IL-1β at this concentration, while it even enhanced the secretion of TNF-α. In accordance to the prediction, paeoniflorin reduced IFN-γ secretion by 29%. Interestingly, it also lowered IL-1β secretion, which was not expected from the results of MVDA. In contrast to the predictions, albiflorin did not reduce IFN-γ secretion, but also reduced IL-1β production. Liquiritigenin strongly lowered the levels of all three cytokines, while isoliquiritigenin did not reduce TNF-α production and also showed weaker effects on IFN-γ production at the selected screening concentration. Astragalosides I and II were predicted to reduce TNF-α and IL-1β secretion and indeed strongly reduced the levels of all three analyzed cytokines. It is worth mentioning that the predictions obtained by OPLS-DA for TNF-α and IL-1β were highly reliable. Concerning IFN-γ, only paeoniflorin and albiflorin could be identified as potentially active compounds. This may be due to the fact, that almost all sub-extracts exerted strong activities against IFN-γ production at the test concentration of 25 µg/mL. Therefore, it seems to be difficult to generate an OPLS-DA model with high quality to make accurate predictions on the basis of the bioassay data that were generated with one screening concentration only. IC_50_ determination and bioactivity classification according to IC_50_ values may lead to the generation of a more meaningful OPLS-DA model in this case. Also, the fact that only one of the two isomers, paeoniflorin, was able to reduce IFN-γ production, depicts the limits of the statistical method to precisely differentiate between structurally similar substances.

These results revealed a panel of compounds within HQJZT, which may contribute to its anti-inflammatory activities. On the other hand, these results confirm the potential of statistical methods combined with LC-MS based metabolomics and pharmacological in vitro assays for prediction and identification of potentially active constituents in complex herbal mixtures. Although their predictive power is not 100 %, the method can be used to narrow down the number of potentially active compounds to be isolated for further investigations. To fully prove the bioactivity of the identified hits, more detailed pharmacological investigations, including the confirmation of dose dependency and IC_50_ determination, are necessary.

## 4. Materials and Methods

### 4.1. Plant Material, Chemicals and Reagents

RA, RC, RRGP, RP, FJ were purchased from Plantasia GmbH—Großhandel für asiatische Heilkräuter (Oberndorf, Austria). Fresh ginger root was bought from Evergreen Agrarprodukte GmbH (Oeynhausen, Austria). Voucher specimens are kept at the Institute of Pharmaceutical Sciences, Department of Pharmacognosy, University of Graz.

The reference substances astragaloside I, astragaloside II, albiflorin, paeoniflorin, calycosin, liquiritigenin and isoliquiritigenin were purchased from PhytoLab GmbH & Co. KG (Vestenbergsgreuth, Germany). 18β-glycyrrhetinic acid and formononetin were purchased from Sigma-Aldrich (St. Louis, MO, USA).

*n*-Hexane (≥95%, for synthesis), dichloromethane (ROTIPURAN^®^ ≥ 99.5% p.a., ACS, ISO), methanol (ROTIPURAN^®^ ≥ 99.9%, p.a., ACS, ISO), ethyl acetate (≥99.5%, Ph. Eur., extra pure) and *n*-butanol (ROTIPURAN^®^ ≥ 99.5% p.a., ACS, ISO) were all purchased from Carl Roth GmbH (Karlsruhe, Germany).

HPLC-grade acetonitrile (HiPerSolv CHROMANORM^®^) was from VWR International SAS (Fontenay Sous Bois, France). Water was purified by a Barnstead EASYpure RF compact ultrapure water system (Thermo Fisher Scientific, Waltham, MA, USA). Formic acid (eluent additive for LC-MS) was purchased from Sigma-Aldrich (St. Louis, MO, USA).

Phosphate buffered saline (PBS), RPMI 1640 medium, heat-inactivated fetal bovine serum (FBS), penicillin, streptomycin, phorbol 12-myristate 13-acetate (PMA), and lipopolysaccharide (LPS) were all from Gibco BRL (Gaithersburg, MD, USA). CCK-8 reagent was bought from Dojindo Molecular Technologies, Inc. (Tokyo, Japan). The ELISA Kit was purchased from eBioscience Inc. (San Diego, CA, USA). DMSO was from Sigma-Aldrich (St. Louis, MO, USA).

### 4.2. Sample Preparation

For preparing classical decoctions, dried plant material of each single herb and of the whole formulation (45 g) was immersed in cold water (360 mL) for 30 min and decocted by boiling for 45 min. The solution was filtered and the procedure was repeated once with the same plant material. Both filtrates were combined and concentrated by a Laborota 4000 rotavapor (Heidolph Instruments, Schwabach, Germany) to approximately 100 mL of extract. Each decoction was transferred to a separation funnel and subsequently extracted with 3 × 30 mL *n*-hexane, dichloromethane, ethyl acetate and *n*-butanol, respectively. These fractions were again concentrated by a rotavapor, dried under nitrogen flow, and stored at 4 °C until use. Since this study focused on the effects of the herbal components in the formula, Saccharum granorum (maltose; added up to 34% of total content in the traditional formula) was not considered in the formula under study.

### 4.3. UPLC-MS Analysis

For UHPLC-HRMS analysis, the dried extracts were dissolved in methanol p.a. in a concentration of 10 mg/mL and filtered through a 0.2 μm syringe filter (RC, Carl Roth GmbH, Karlsruhe, Germany). The reference compounds were dissolved at a concentration of 1 mg/mL in methanol p.a.

UPLC-QTOF-MS analysis was carried out using an ACQUITY UPLC^®^ I-Class system with a flow-through needle and a column heater (Waters Corporation, Milford, MA, USA). The instrument was interfaced with a Xevo^®^ G2-S QTOF quadrupole time-of-flight mass spectrometer that was run in the ESI positive mode. The acquisition range was at 100-1800 Da with a scan time of 0.1 sec, gas flow 10 L/h (cone gas) and 800 L/h (desolvation gas), collision energy 6 eV (low CE), 15-45 eV ramping (high CE), source heater temperature 120 °C, desolvation temperature 500 °C, capillary voltage 3.0 kV and cone voltage 30 V. The separation was carried out on a reversed-phase column (ACQUITY UPLC HSS T3, 1.8 µm, 2.1 × 100 mm) at 40 °C. As a mobile phase, a gradient of 0.1% formic acid in water (A) and 0.1% formic acid in acetonitrile (B) was used at a flow rate of 0.5 mL/min; 5 µL of each sample were injected. The gradient profile was optimized as follows: 0 min: A–B 95:5 (*v*/*v*); 0.5 min: A–B 99:1 (*v*/*v*); 15 min: A–B 60:40 (*v*/*v*); 40 min: A–B 0:100 (*v*/*v*); 44 min: A–B 0:100 (*v*/*v*); 45 min: A–B 95:5 (*v*/*v*). Three replicate injections per sample were performed [4,7].

### 4.4. Pharmalogical Studies

U937 cells were obtained from the American Type Culture Collection (Rockville, MD, USA) and cultured in RPMI 1640 medium supplemented with 10% heat-inactivated FBS, 100 U/mL penicillin and 100 mg/mL streptomycin at 37 °C in a humidified atmosphere with 5% CO_2_. Stock solutions of all extracts in DMSO (20 mg/mL) were prepared and further diluted to desired concentrations with PBS buffer. In order to determine a concentration that is non-toxic to the leukemic cells for further investigations, all samples were tested at different concentrations (3.125, 6.25, 12.5, 25, 50 and 100 µg/mL) using the CCK-8 (Cell Counting Kit-8) cell viability assay. The U937 cells were seeded into 96 well plates (1.0 × 105 cells/mL) and incubated with 10 ng/mL PMA for 48 h at 37 °C in a 5% CO_2_-humidified atmosphere to induce differentiation of macrophages. Afterwards, the cells were washed three times with PBS buffer to remove remaining PMA and non-adherent cells. The cells were incubated with the samples for 21 h. at 5% CO_2_ and 37 °C. 10 μL CCK-8 reagent were added to each well and the cells were incubated for further three hrs. Finally, the absorbance of each well was measured using a microplate reader at 450 nm.

For determination of inhibitory activity on cytokine production, U937 cells were seeded into six well plates (1.0 × 106 cells/mL) and incubated with 10 ng/mL PMA for 48 h. at 37 °C in a 5% CO_2_-humidified atmosphere to induce differentiation of macrophages. The medium was changed and the cells were pretreated with extracts or pure compounds (final concentration 25 µg/mL and 6.25 µg/mL, respectively) for two hrs and then incubated for another 24 h with or without 1000 ng/mL LPS. Afterwards, the culture supernatants were collected and the levels of TNF-α, IL-1β and IFN-γ were determined by the human TNF-α, IL-1β and IFN-γ Platinum ELISA, following the manufacturer’s instructions [4,7].

### 4.5. Data Processing and Multivariate Data Analysis

UPLC-HRMS data were processed with UNIFI software (Waters Corporation, Milford, MA, USA), resulting in a data matrix that consisted of all features detected in each sample (i.e., m/z-retention time pairs) and their respective peak areas. After evaluation of the UPLC-MS raw data, peaks detected after min 33 were found to be artifacts, since they also were detected in the blank runs with methanol. Therefore, only features detected from 0–33 min were included for statistical analysis. The data matrix was exported to Microsoft Excel (Microsoft Corporation, Redmond, WA, USA), and every peak was normalized to the total area of all peaks between 0 and 33 minutes in the respective run. Then, the data were log transformed, Pareto scaled, and subjected to multivariate data analysis by using SIMCA 13.0.3 software (Umetrics, Umea, Sweden) (Supplementary Materials). Orthogonal Projection of Latent Structures Discriminant Analysis (OPLS-DA) was performed in order to determine the metabolic variables (i.e. the UPLC-HRMS features) associated with observed in vitro pharmacological activity. For OPLS-DA, the results from the pharmacological assays were classified into three groups (1 = active, 2 = moderately active, and 3 = inactive, see Table 1) and correlated to the processed UPLC-QTOF-MS dataset. Group 1 and group 3 (i.e., active and inactive samples) were included in the OPLS-DA models generated for each of the three tested bioactivities (i.e. TNF-α, IL-1β and IFN-γ secretion). From the S-plots derived from the OPLS-DA models, 18–20 compounds most likely associated with the observed bioactivity (OPLS-DA hits) were selected.

### 4.6. Metabolite Identification

Targeted identification of the OPLS-DA hits was performed on the one hand by Traditional Medicine Library (Waters Corporation, Milford, MA, USA) included in the UNIFI data processing software on the basis of precursor exact mass, fragment ion mass, and theoretical isotopic distribution, and on the other hand by comparison with existing data from literature and databases. Tentative identification was verified by UHPLC-MS comparison with authentic reference standards when available.

## 5. Conclusions

In this study, we performed pharmacological in vitro tests on the inhibitory activity of HQJZT decoction on the production of the cytokines TNF-α, IL-1β and IFN-γ in U937 human leukemic cells in order to identify potentially active constituents and to substantiate the traditional use of the formulation in chronic gastrointestinal inflammation. The results of the pharmacological screening indicate potential anti-inflammatory and immunomodulatory effects of the formula and its individual herbal components. Correlating the pharmacological results to UPLC-HRMS metabolomics data allowed us to predict the constituents most likely contributing to the observed activities. Overall, 16 compounds were tentatively identified, seven of which, namely formononetin, calycosin, astragaloside I, liquiritigenin, 18ß-glycyrrhetinic acid, paeoniflorin, and albiflorin, were unambiguously identified by LC-MS comparison with reference substances. Literature data confirmed that these compounds possess anti-inflammatory activities. The findings from the statistical predictions were verified in cellular cytokine production assays with the identified pure compounds. Astragaloside I, astragaloside II, calycosin, formononetin, liquiritigenin, and paeoniflorin were all proven to exert anti-inflammatory activities in in vitro cytokine production inhibition assays.

With these findings, we could partially verify the predictions of the OPLS-DA model and substantiate the anti-inflammatory activity of HQJZT by identifying constituents involved in the observed bioactivities, as well as the usefulness and limitations of MVDA for the prediction of active compounds. Due to the fast developments in analytical technologies, data acquisition is becoming easier, leading to huge amounts of high-resolution data. HR-LC-MS combined with multivariate statistical analysis, like PCA and OPLS-DA, proved to be an effective tool for the determination and identification of the constituents that are potentially related to the bioactivity of a complex herbal formula. Further studies to fully prove the bioactivity of the hit compounds that has only been shown on the basis of one screening concentration in the present study will be necessary in the future.

## Figures and Tables

**Table 1:** Classification of the pharmacological activities.

Activity Class	TNF-α Inhibition (%)	IL-1β Inhibition (%)	IFN-γ Inhibition (%)
1 (active)	70–100	60–100	60–100
2 (moderately active)	50–69	45–59	45–59
3 (inactive)	0–49	0–44	0–44

**Table 2:** Summary of all tentatively identified hit compounds from the three tested activities.

RT (min)	Detected *m*/*z* (ESI+)	(Tentative) Identification	Origin	Mono-Isotopic Mass (g/mol)	Molecular Formula	MS/MS Fragment Ions	Literature for Identification	Pharmacological Activity Predicted by OPLS-DA	p[1]	po[1]
4.82	481.17023	Albiflorin *	RP	480.163	C23H28O11	197, 151, 133	[22,23]	IFN-γ	−0.0650068	−0.0111313
5.34	503.15227	Paeoniflorin * (Na-Adduct)	RP	480.163	C23H28O11	179, 151, 133	[22,23]	IFN-γ	−0.0719782	−0.0112909
6.48	447.1285	Calycosin-7-*O*-β-d-glucoside or isomer	RA	446.121	C22H22O10	270, 253, 225, 137	[24]	TNF-α	−0.0687443	0.132943
8.39	503.1522	Albiflorin isomer (Na-Adduct)	RP	480.163	C23H28O11	219, 197, 133, 105	[22,23]	IFN-γ	−0.0562873	−0.0109413
9.82	257.08161	Liquiritigenin *	RRGP	256.074	C15H12O4	137, 147, 119, 261, 91, 81, 215, 159		TNF-α IL-1β	−0.0535154 −0.0652777	−0.0175027 −0.0002341
9.98	464.2489	Pinen-10-yl-ß-vicianoside (NH4-Adduct)	RP	446.215	C21H34O10	133, 127, 115		IFN-γ	−0.0438006	−0.005821
10.01	301.1075	3-Hydroxy-9,10-dimethoxypterocarpan or isomer	RA	300.1	C17H16O5	269, 191, 167, 152, 147, 133, 123, 105	[25]	TNF-α IL-1β	−0.0767608 −0.0794242	0.176659 0.113695
10.51	285.07656	Calycosin *	RA	284.068	C16H12O5	270, 253, 225, 229, 241, 137	[24,25]	TNF-α, IL-1β	−0.151342 −0.167255	0.247407 0.258227
10.94	315.0866	Odoratin	RA	314.079	C17H14O6	300, 283, 259, 255, 244, 167	Metlin	IL-1β	−0.0583033	0.116079
13.77	269.08165	Formononetin *	RA	268.074	C16H12O4	237, 209, 213, 225, 136	Metlin, [24]	TNF-α IL-1β	−0.135887 −0.153853	0.108774 0.218006
14.15	301.1077	3-Hydroxy-9,10-dimethoxypterocarpan or isomer	RA	300.1	C17H16O5	269, 191, 167, 152, 147, 133, 123, 105	[25]	TNF-α IL-1β	−0.0535515 −0.0733961	0.0891372 0.166495
15.87	1653.9439	Astragaloside II (dimer)	RA	798.44	C41H66O15	647, 629, 491, 473, 455, 437, 419	[24,26]	TNF-α	−0.0558838	0.154538
18.39	1737.9663	Astragaloside I * (dimer)	RA	868.482	C45H72O16	689, 671, 653, 491, 473, 455, 437, 419, 297	[24,26]	TNF-α IL-1β	−0.0831685 −0.0743063	0.219044 0.1784
19.15	1737.9633	Astragaloside I isomer (dimer)	RA	868.482	C45H72O16	671, 653, 491, 473, 455, 437, 419, 297	[24,26]	TNF-α IL-1β	−0.0827036 −0.0743328	0.216071 0.178967
20.22	1737.9632	Astragaloside I isomer (dimer)	RA	868.482	C45H72O16	851, 833, 689, 671, 653, 491, 473, 455, 437, 419, 297	[24,26]	TNF-α	−0.0571481	0.146308
24.71	471.3464	18β-Glycyrrhetinic acid *	RRGP	470.34	C30H46O4	453, 235, 217, 189, 175, 189	Metlin	TNF-α	−0.0542538	−0.0348534

Abbreviations: RP-Radix Paeoniae Alba; RA-Radix Astragali; RRGP-Radix et Rhizoma Glycyrrhizae Praparata cum Melle; * unambiguously identified by comparison with authentic reference compound.

## Supplementary Materials

The data are available online at https://www.mdpi.com/1420-3049/24/17/3116/s1, Table S1: UHPLC-MS/MS features used for data analysis in SIMCA.

## Appendix A

**Table A1:** Extract Yields obtained by liquid-liquid extraction of HQJZT and its single herbal components.

Plant	Initial Weight Crude Drug (g)	Yield n-Hexane Extract (mg)	Yield DCM Extract (mg)	Yield EtOAc Extract (mg)	Yield n-Butanol Extract (mg)	Yield Water Residue (g)
Huangqin Jianzhong Tang	45	34.1	63.6	209.0	962.9	7.8
Radix Astragali	45	12.3	93.6	104.6	803.6	13.7
Ramulus Cinnamomi	20	33.7	56.1	51.3	161.6	1.0
Radix et Rhizoma Glycyrrhizae praeparata cum melle	20	22.4	46.0	147.3	609.2	6.8
Fructus Jujubae	45	19.4	28.2	45.1	178.9	8.6
Radix Paeoniae albae	20	19.8	43.2	245.9	723.7	3.7
Rhizoma Zingiberis recens	45	39.3	17.0	6.3	55.4	1.0

**Table A2:** Hits for the inhibitory activity of HQJZT against TNF-α production.

*m*/*z*_RT	p[1]	po[1]	Origin	Compound	Fragment Ions	Literature
285.07656_10.514	−0.151342	0.247407	RA	Calycosin *	253, 225, 137	[24,25]
269.08165_13.771	−0.135887	0.108774	RA	Formononetin *	269, 253, 237, 226, 197, 181, 169, 137, 118	Metlin, [26]
1737.96629_18.398	−0.0831685	0.219044	RA	Astragaloside I * (dimer)	689, 671, 653, 491, 473, 455, 437, 419, 297	[24,26]
1737.96335_19.162	−0.0827036	0.216071	RA	Astragaloside I isomer (dimer)	689, 671, 653, 491, 473, 455, 437, 419, 297	[24,26]
355.11759_16.953	−0.0798714	−0.0373342	RRGP	n.i.		
301.10751_10.011	−0.0767608	0.176659	RA	3-Hydroxy-9,10-dimethoxypterocarpan or isomer	269, 191, 167, 152, 133, 147, 123, 105	[25]
371.10109_32.627	−0.0708791	0.069922	All	n.i.		
447.12852_6.654	−0.0687443	0.132943	RA	Calycosin-7-*O*-β-d-glucoside or isomer	270, 253, 225, 137	[24]
355.06981_32.607	−0.0640716	0.124033	RA	n.i.		
714.41099_18.603	−0.0632131	−0.0573972	RZ	n.i.		
411.22785_13.841	−0.0617407	−0.0464029	RZ	n.i.		
508.25367_16.894	−0.0592386	−0.0425658	RZ	n.i.		
438.23858_13.465	−0.0591264	−0.0471358	RZ	n.i.		
447.12854_6.484	−0.0589284	0.116497	RA	Calycosin-7-*O*-β-d-glucoside or isomer	270, 253, 225, 137	[24]
1737.96324_20.222	−0.0571481	0.146308	RA	Astragaloside I isomer (dimer)	851, 833, 689, 671, 653, 491, 473, 455, 437, 419, 297	[24,26]
1653.94391_15.879	−0.0558838	0.154538	RA	Astragaloside II or isomer (dimer)	647, 629, 491, 473, 455, 437, 419	[24,26]
353.10204_16.488	−0.0557885	−0.0258396	RRGP	n.i.		
471.34640_24.718	−0.0542538	−0.0348534	RRGP	18β-Glycyrrhetinic acid *	453, 235, 217, 189, 175189	Metlin
301.10773_14.151	−0.0535515	0.0891372	RA	3-Hydroxy-9,10-dimethoxypterocarpan or isomer	269, 191, 167, 152, 147, 133, 123, 105	[25]
257.08161_9.822	−0.0535154	−0.0175027	RRGP	Liquiritigenin *	261, 215, 159, 147, 137, 119, 91, 81	

* Identified by comparison with a reference compound; RA = Radix Astragali; RC = Ramulus Cinnamomi; RRGP = Radix et Rhizoma Glycyrrhiza praep cum melle; FJ = Fructus Jujuba, RP = Radix Paeoniae alba; RZ = Rhizoma Zingiberis recens.

**Table A3:** Hits for the inhibitory activity of HQJZT against IL-1β production.

*m*/*z* RT	p[1]	po[1]	Origin	Compound	Fragment Ions	Literature
285.07656_10.514	−0.167255	0.258227	RA	Calycosin *	270, 253, 241, 229, 225, 137	[24,25]
269.08165_13.771	−0.153853	0.218006	RA	Formononetin *	237, 225, 213, 209, 136	Metlin, [24]
355.11759_16.953	−0.0865657	−0.002337	RRGP	n.i.		
714.41099_18.603	−0.0851547	−0.0579323	RZ	n.i.		
301.10751_10.011	−0.0794242	0.113695	RA	3-Hydroxy-9,10-dimethoxypterocarpan	269, 191, 167, 152, 147, 133, 123, 105	[25]
1737.96335_19.162	−0.0743328	0.178967	RA	Astragaloside I isomer (dimer)	851, 833, 689, 671, 653, 491, 473, 455, 437, 419	[24,26]
1737.96629_18.398	−0.0743063	0.1784	RA	Astragaloside I * (dimer)	851, 833, 689, 671, 653, 491, 473, 455, 437, 419	[24,26]
301.10773_14.151	−0.0733961	0.166495	RA	3-Hydroxy-9,10-dimethoxypterocarpan	269, 191, 179, 167, 152, 133, 147, 123, 105	[25]
371.10109_32.627	−0.0708126	0.0542131	All	n.i.		
508.25367_16.894	−0.0670202	−0.0193137	RZ	n.i.		
355.06981_32.607	−0.0669833	0.135838	RA	n.i.		
891.46950_19.156	−0.0653422	0.0481451	RA	Astragaloside I isomer	671, 653, 491, 473, 455, 437, 419, 297	[24,26]
257.08161_9.822	−0.0652777	−0.0002341	RRGP	Liquiritigenin *	261, 215, 159, 147, 137, 119	
891.47006_18.395	−0.0652695	0.0662892	RA	Astragaloside I * [M + Na]^+^	689, 671, 653, 491, 473, 455, 437, 419, 297	[24,26]
447.12852_6.654	−0.0627192	−0.0564573	RA	Calycosin-7-*O*-β-d-glucoside	270, 253, 225, 137	[24]
353.10204_16.488	−0.059812	0.0144688	All	n.i.		
473.14387_12.038	−0.0597473	0.0753214	RA	n.i.		
315.08661_10.947	−0.0583033	0.116079	RA	Odoratin	300, 283, 259, 255, 244, 167	Metlin

* Identified by comparison with a reference compound; RA = Radix Astragali; RC = Ramulus Cinnamomi; RRGP = Radix et Rhizoma Glycyrrhiza praep cum melle; FJ = Fructus Jujuba, RP = Radix Paeoniae alba; RZ = Rhizoma Zingiberis recens.

**Table A4:** Hits for the inhibitory activity of HQJZT against IFN-γ production.

*m*/*z* RT	p[1]	po[1]	Origin	Compound	Fragment Ions	Literature
265.12345_8.988	−0.0930574	0.175785	FJ	n.i.		
481.17020_5.056	−0.0759404	−0.013529	RP	Albiflorin *	503 [M + Na], 197, 151, 133	[22,23]
415.21121_18.824	−0.0736124	0.0616802	FJ	n.i.		
503.15230_5.509	−0.0722717	−0.0109856	RP	Paeoniflorin * [M + Na]^+^	498 [M + NH4], 179, 151, 133	[22,23]
503.15227_5.348	−0.0719782	−0.0112909	RP	Paeoniflorin * [M + Na]^+^	498 [M + NH4], 179, 151, 133	[22,23]
498.19688_5.504	−0.0712826	−0.0135778	RP	Paeoniflorin * [M + NH4]^+^	503 [M + Na], 179, 151, 133	[22,23]
481.17023_4.821	−0.0650068	−0.0111313	RP	Albiflorin *	503 [M + Na], 197, 151, 133	[22,23]
714.41099_18.603	−0.0639971	0.0657522	RZ	n.i.		
452.32137_22.770	−0.0626265	0.0403574	FJ	n.i.		
503.15215_5.060	−0.0608256	−0.0112778	RP	Albiflorin * [M + Na]^+^	498 [M + NH4], 197, 151, 133	[22,23]
498.19685_5.348	−0.0600451	−0.0104305	RP	Paeoniflorin *	503 [M + Na], 179, 151, 133	[22,23]
503.15220_8.397	−0.0562873	−0.0109413	RP	Albiflorin isomer	219, 197, 133, 105	[22,23]
282.14928_9.203	−0.0533495	0.0556512	FJ	n.i.		
503.15228_4.813	−0.0531328	−0.0098537	RP	Albiflorin * [M + Na]^+^	498 [M + NH4], 197, 151, 133	[22,23]
438.23858_13.465	−0.0501564	0.0505945	RZ	n.i.		
983.31365_5.058	−0.0496619	−0.0078355	RP	Albiflorin *	503 [M + Na], 481, 197, 151, 133, 105	[22,23]
379.24752_22.864	−0.0443945	0.0688323	FJ	n.i.		
464.24899_9.989	−0.0438006	−0.005821	RP	Pinen-10-yl-ß-vicianoside	133, 127, 115	
265.12281_9.203	−0.0422487	0.010489	RZ	n.i.		
983.31391_4.812	−0.040604	−0.0057421	RP	Albiflorin *	503 [M + Na], 481, 197, 151, 133, 105	[22,23]

* Identified by comparison with a reference compound; RA = Radix Astragali; RC = Ramulus Cinnamomi; RRGP = Radix et Rhizoma Glycyrrhiza praep cum melle; FJ = Fructus Jujuba, RP = Radix Paeoniae alba; RZ = Rhizoma Zingiberis recens.

**Table A5:** Summary of the predicted and verified effects of the tested pure hit compounds, at a screening concentration of 6.25 µg/mL.

Compound	Chemical Structure	Predicted Activity	Verified Activity
Astragaloside I		TNF-α, IL-1β	TNF-α, IL-1β, IFN-γ
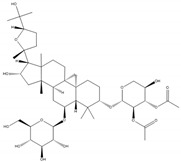
Astragaloside II		TNF-α	TNF-α, IL-1β, IFN-γ
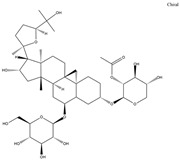
Calycosin		TNF-α, IL-1β	IL-1β
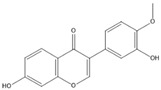
Formononetin		TNF-α, IL-1β	TNF-α, IL-1β, IFN-γ
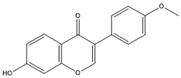
Liquiritigenin		TNF-α, IL-1β	TNF-α, IL-1β, IFN-γ
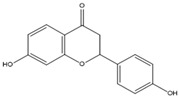
Albiflorin		IFN-γ	IL-1β,
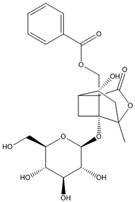
Paeoniflorin		IFN-γ	IL-1β, IFN-γ
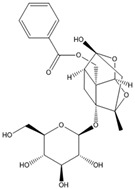
Isoliquiritigenin			IL-1β, IFN-γ
